# Operator radiation burden and periprocedural outcomes in robotic-assisted versus manual percutaneous coronary intervention: a meta-analysis

**DOI:** 10.1186/s12893-026-03863-7

**Published:** 2026-05-22

**Authors:** Yaxu Xin, Linyun Wang, Fei Han, Qingning Chang, Chengze Li, Chao Ding, Jingyi Xu, Ruoyu Wang, Yiru Wang, Kaiyong Wang, Yupei Dai, Guohang Shen, Feng Li

**Affiliations:** 1 The First Clinical Medical College, Ningxia Medical University, Yinchuan, Ningxia 750004 PR China; 2 Center of Laboratory Medicine, General Hospital of Ningxia Mediccal University, 804 Shengli South Street, Yinchuan, Ningxia 750004 PR China; 3 North Sichuan Medical College, No.234 Fujiang Road, Shunqing District, Nanchong, 637000 Sichuan China

**Keywords:** Robotic-assisted percutaneous coronary intervention, Manual percutaneous coronary intervention, Operator radiation exposure, Postprocedural outcomes

## Abstract

**Background:**

Robotic-assisted percutaneous coronary intervention (rPCI) was introduced in part to reduce occupational radiation exposure and improve ergonomics in the catheterization laboratory, while also offering potential procedural advantages. However, the comparative clinical value of rPCI versus manual PCI (mPCI) remains uncertain. This meta-analysis aimed to evaluate the relative effects of rPCI and mPCI on postprocedural outcomes, healthcare personnel radiation exposure, postprocedural complications, and follow-up outcomes.

**Methods:**

A systematic search was conducted in PubMed, Embase, Web of Science, and the Cochrane Library to identify eligible studies published from January 2014 to January 2026. After rigorous study selection, data extraction, and methodological quality assessment, quantitative synthesis was performed using RevMan 5.4. Odds ratios (ORs) were used for dichotomous outcomes and mean differences (MDs) for continuous outcomes. A random-effects model was applied when heterogeneity was significant (I²>50%); otherwise, a fixed-effects model was used.

**Results:**

Compared with mPCI, rPCI was associated with a consistent reduction in operator radiation exposure. Patient- and procedure-related radiation indices, including dose-area product, air kerma, and fluoroscopy time, also tended to favor rPCI. Procedural duration was longer with rPCI, whereas contrast use was comparable between groups. rPCI was also associated with higher clinical success and postprocedural TIMI 3 flow, while technical success and coronary dissection rates were similar between groups. At 12-month follow-up, rPCI showed a trend toward lower rates of major adverse cardiovascular events and all-cause mortality.

**Conclusions:**

The available data support a clear occupational safety advantage of rPCI and suggest favorable periprocedural safety and efficacy profiles, with potential clinical benefits. However, these findings, particularly for patient-level outcomes, should be considered hypothesis-generating rather than conclusive, as they are primarily derived from observational studies with limited sample sizes and require confirmation in larger randomized trials.

**Supplementary Information:**

The online version contains supplementary material available at 10.1186/s12893-026-03863-7.

## Introduction

Percutaneous coronary intervention (PCI) has become a standard treatment for obstructive coronary artery disease in both stable and acute clinical settings [1, 2]. Despite these advances, the basic operating model of PCI has changed relatively little, and concerns about operator radiation exposure and ergonomic strain remain unresolved [3–5]. In routine manual PCI (mPCI), the operator typically works at the procedure table under fluoroscopic guidance and relies on personal and structural shielding for radiation protection [3, 6]. This working environment exposes interventional cardiologists to repeated ionizing radiation and to the physical burden of prolonged lead use, both of which have well-recognized occupational consequences [7, 8]. Reports of cataracts, orthopedic injury, and other radiation- or practice-related health risks have made operator safety an increasingly important issue in contemporary interventional cardiology [9–11]. Robotic-assisted PCI (rPCI) was introduced in this context. By allowing the primary operator to perform the interventional portion of the procedure from a radiation-shielded cockpit, robotic systems were developed largely to reduce occupational radiation exposure and improve ergonomics. Early studies showed that robotic PCI was technically feasible and could be performed safely in selected coronary lesions, while substantially reducing operator radiation exposure [10, 12, 13].

In addition to operator protection, robotic platforms may offer procedural advantages. Their ability to manipulate devices in small increments and to facilitate more objective lesion-length measurement has raised the possibility that rPCI may improve stent positioning and reduce longitudinal geographic miss, which has been associated with restenosis and repeat revascularization [14–16]. As experience with robotic systems has expanded, the focus of the field has shifted from feasibility to comparative effectiveness. The central question is no longer whether rPCI can be performed, but how it compares with mPCI in routine clinical practice. Available comparative studies have provided encouraging but not entirely consistent results [17–19]. Comparative studies have generally reported high procedural and clinical success rates with rPCI, including in more complex lesions, although findings for other outcomes have been less consistent. Some studies have suggested lower patient radiation exposure with rPCI, whereas others have not demonstrated a clear advantage [18, 20]. Similarly, fluoroscopy time, procedural duration, contrast use, and patient radiation metrics have not been reported consistently across studies. For example, a large propensity-matched study suggested lower patient radiation exposure with rPCI, but longer procedural duration and no significant difference in fluoroscopy time or contrast volume, whereas another matched cohort study reported longer fluoroscopy time with rPCI and no clear advantage in patient radiation-related measures [17–19].

The uncertainty is partly related to the structure of the existing evidence. Much of the literature consists of single-center observational studies, often retrospective or propensity matched, rather than randomized comparisons [19]. Study populations also differed substantially across studies. Some investigations focused on selected elective lesions or narrowly defined procedural subsets, whereas others enrolled patients with more complex anatomy or broader contemporary practice cohorts [13, 18]. In parallel, robotic technology has evolved from first- to second-generation systems, and outcomes may reasonably be influenced by differences in platform capability, operator experience, and workflow adaptation [20–22]. These differences make it difficult to determine whether between-study variation reflects the true effect of robotic assistance or differences in case selection and study design [13, 14, 18]. Another unresolved issue is the clinical value of rPCI from the patient perspective. The most consistent advantage of robotic PCI has been operator benefit, whereas wider adoption will depend on whether this technology can preserve procedural efficiency and achieve clinical outcomes at least comparable to those of conventional PCI [19, 20]. Existing studies have addressed this question, but most have been underpowered for harder clinical endpoints, and follow-up data remain relatively limited [11, 19]. In view of these uncertainties, a systematic review and meta-analysis may help clarify how rPCI compares with mPCI in current practice. Quantitative synthesis may help better define the procedural and clinical profile of rPCI relative to mPCI and clarify which findings are consistent across studies and which remain uncertain [18, 23]. Therefore, the present study compared robotic-assisted and manual PCI with respect to procedural characteristics and clinical outcomes.

## Methods

### Literature search strategy

This systematic review and meta-analysis was conducted in accordance with the PRISMA guidelines and prospectively registered on PROSPERO (registration ID: CRD420261345511) [24]. A comprehensive search of PubMed, Embase, Web of Science, and the Cochrane Library was performed to identify eligible studies published up to January 2026. The search strategy combined keywords and Medical Subject Headings (MeSH), including “manual,” “robot-assisted,” and “percutaneous coronary intervention.” Only English-language publications were included. References from the identified clinical studies as well as meta-analyses were reviewed by hand to identify further relevant reports. Two reviewers independently screened all records for eligibility. Two reviewers also independently extracted study characteristics, baseline variables, and outcome data using a standardized data collection form; discrepancies were resolved by discussion and, when necessary, adjudication by a third reviewer. To avoid duplicate inclusion from overlapping cohorts, the most complete or most recent report was retained. Boolean operators (AND/OR) were applied to broaden the search, and multiple combinations of keywords were used repeatedly.

### Inclusion criteria and data extraction

Inclusion criteria were defined according to the PICOS framework (Population, Intervention, Comparison, Outcomes, and Study design): Population (P): Patients with coronary artery disease undergoing PCI. Intervention (I): Robotic-assisted percutaneous coronary intervention (rPCI). Comparison (C): Manual percutaneous coronary intervention (mPCI). Outcomes (O): Postprocedural outcomes, postprocedural cardiovascular complications, operator radiation exposure. Study design (S): Randomized controlled trials (RCT), prospective observational studies (PCS), and retrospective observational studies (RCS) [25]. All included studies evaluated rPCI and mPCI for the management of coronary artery disease, with no other interventional procedures involved in the analysis. Thus, the pooled estimates primarily reflect the clinical outcomes of cohorts undergoing rPCI or mPCI for coronary artery disease. The extracted data comprised demographic and baseline clinical variables, cardiovascular comorbidities, smoking history, and coronary lesion characteristics. Exclusion criteria were duplicate publications, non-comparative designs (including case reports, reviews, conference abstracts, meta-analyses, or single-arm studies), non-English articles, animal or in vitro studies, and studies with insufficient data for effect-size extraction. Study quality was not used as an a priori exclusion criterion; instead, methodological quality and risk of bias were assessed formally and considered in sensitivity analyses. Since there is only one RCT and all others are observational studies, we combined randomized and non-randomized studies to maximize the available evidence, especially in the analysis of operator-related outcomes and rare events. While this approach introduces methodological heterogeneity, we performed sensitivity analyses and assessed study quality using ROBINS-I and NOS to mitigate potential bias. We acknowledge that differences in study design, risk of bias, and baseline characteristics may still influence pooled estimates.

In total, 12 outcome indicators were analyzed. These were prespecified as follows: Primary outcomes included technical success rate, clinical success rate, post-procedural TIMI 3 flow rate, major adverse cardiovascular events (MACE), post-procedural coronary dissection, and 12-month all-cause mortality. The specific components of MACE in each included study are summarized in Supplementary Table 1. As secondary outcomes, we assessed procedural duration, fluoroscopy time, contrast volume, dose-area product (DAP), radiation air kerma (RAK), and operator radiation exposure. We also reviewed how key outcomes were defined and recorded across studies to better interpret between-study variability. In most studies, procedural duration was reported as the total procedural duration from vascular access puncture to sheath removal, in minutes; fluoroscopy time was defined as the cumulative X-ray exposure time during the entire PCI procedure, in minutes; contrast volume was recorded as the total volume of iodinated contrast medi

um administered intraoperatively, in milliliters. Radiation-related outcomes were uniformly quantified with DAP expressed in dGy·cm², RAK in mGy, and operator radiation exposure in microsieverts (µSv). Clinical efficacy endpoints (technical success rate, clinical success rate, post-procedural TIMI 3 flow rate) and safety outcomes (MACE, post-procedural coronary dissection, 12-month all-cause mortality) were reported as categorical variables with proportions calculated based on the total number of patients in each study group. Although some outcome definitions and recording times varied across studies, the overall reporting patterns were broadly comparable and likely explain part of the observed heterogeneity.

### Quality assessment, publication bias, and statistical analysis

The Newcastle–Ottawa Scale (NOS) was used to evaluate methodological quality, with scores ≥ 7 considered acceptable and 8–9 classified as high quality. For non-randomized studies, risk of bias was additionally assessed using the ROBINS-I tool, covering seven domains (confounding, participant selection, classification of interventions, deviations from intended interventions, missing data, outcome measurement, and reporting) [26]. Each domain was judged as low, moderate, serious, or critical, with the overall rating determined by the highest level of risk; studies rated as serious or critical were regarded as low quality and included in sensitivity analyses. For studies reporting medians and interquartile ranges, means and standard deviations were estimated using the methods of Luo, McGrath, and Shi [27–29]. A minority of continuous outcomes required such conversion. Sensitivity analyses indicated that these transformations had minimal impact on heterogeneity patterns or pooled effect estimates. Pooled effect sizes were expressed as ORs for binary outcomes and MDs for continuous outcomes, both with 95% confidence intervals CIs. Heterogeneity was assessed using the I² statistic, with values of 25%, 50%, and 75% generally considered to represent low, moderate, and high heterogeneity, respectively, and sensitivity analyses were performed by excluding influential studies or those with a higher risk of bias [30, 31]. Primary eligibility criteria and core analytic rules followed the registered PROSPERO protocol. Additional exploratory post hoc subgroup and sensitivity analyses were performed to investigate marked heterogeneity in selected outcomes. These analyses were not prespecified in the PROSPERO protocol and should be considered exploratory. Statistical analyses were performed using Review Manager (RevMan, version 5.4; Cochrane Collaboration, Oxford, UK). A two-sided P value < 0.05 was considered statistically significant. The analytic approach was reviewed by a professional biostatistician [32, 33]. The study-level extracted values and the computation files (RevMan project file) have been provided in the supplementary materials to facilitate reproducibility.

## Results

### Study selection and characteristics

**Fig. 1:**
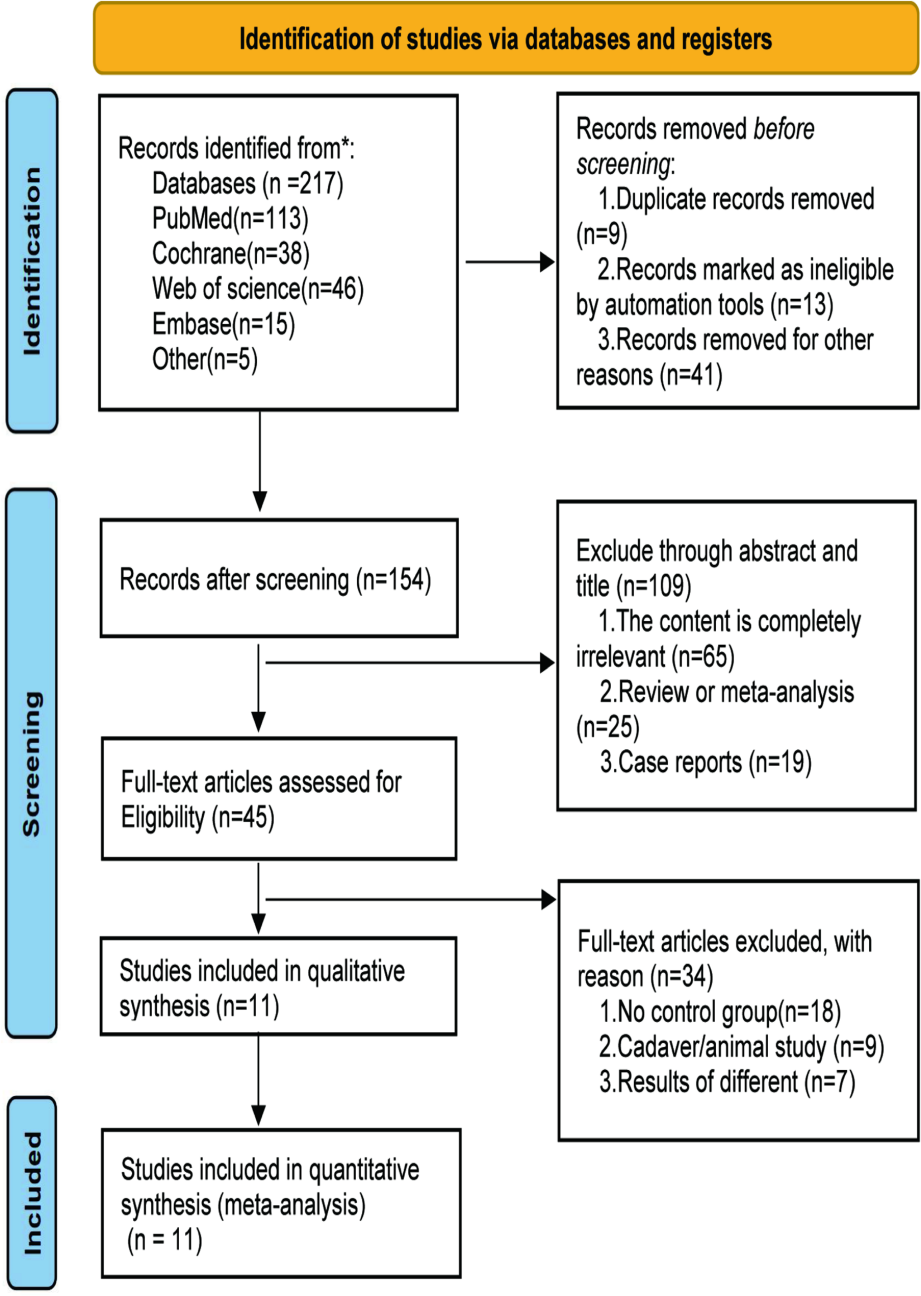
PRISMA flow diagram of study selection

A total of 217 articles were identified through database and manual searches. After full-text screening (Fig. 1 shows the flow diagram of study selection), 11 articles reporting 12 outcome indicators were included in the final analysis, encompassing 2,950 patients. Of these, 974 patients underwent rPCI and 1,976 underwent mPCI [9–15, 17–19, 34]. Table 1 summarizes the baseline characteristics of the included studies. Overall, the rPCI and mPCI groups were broadly comparable with respect to age, sex, body mass index, smoking history, cardiovascular comorbidities, and lesion length. Table 2 provides an overview of the 12 outcome measures. Primary outcomes included technical success rate, clinical success rate, post-procedural TIMI 3 flow rate, post-procedural coronary dissection, and 12-month follow-up outcomes including MACE and all-cause mortality. As secondary outcomes, we assessed procedural duration, fluoroscopy time, contrast volume, DAP, RAK, and operator radiation exposure.

**Table 1:** Main characteristics of the included studies

Study ID (Year)	Group	Cases(*n*)	Study design	Age, years (mean ± SD)	Female/Male (*n*)	BMI, kg/m² (mean ± SD)	Arterial hypertension *n* (%)	History of smoking *n* (%)	Diabetes mellitus *n* (%)	Lesion length, mm	Radial artery approach, *n* (%)
Bay 2024 [18]	rPCI	85	PCS	71.6 ± 7.1	23/62	26.4 ± 3.6	74(87.1)	17(20)	16(18.6)	24.1 ± 19.7	64(75.7)
	mPCI	226		70.7 ± 5.3	54/172	26.9 ± 3.5	198(87.6)	41(18.1)	20(9)	19.1 ± 14.8	140(61.9)
Hirai 2020 [34]	rPCI	49	RCS	65.5 ± 9.2	10/39	29.0 ± 4.3	NR	NR	21(42.9)	NR	NR
	mPCI	46		63.6 ± 12.5	9/37	30.7 ± 5.9			20(43.5)		
Kagiyama 2021 [9]	rPCI	30	RCS	70.9 ± 9.9	7/23	NR	22(75.0)	16(53.3)	NR	15.2 ± 7.7	18(60.4)
	mPCI	77		73.0 ± 11.3	29/48		66(86.3)	31(42.5)		14.5 ± 9.4	42(54.1)
Madder 2016 [10]	rPCI	45	PCS	NR	19/36	NR	NR	NR	NR	NR	38(84.4)
	mPCI	168			80/88						113(67.1)
Mahmud 2017 [13]	rPCI	108	RCS	68 ± 11	24/84	NR	114(95)	NR	61(56)	22.2 ± 10.6	13(12)
	mPCI	226		67 ± 12	50/176		215(95)		122(54)	19.4 ± 9.5	28(12.4)
Mangels 2020 [15]	rPCI	56	RCS	68.9 ± 10.8	11/45	NR	45(80.5)	40(71.4)	16(28)	NR	NR
	mPCI	108		66.5 ± 9.5	21/87		90(83.2)	50(46.2)	38(35)		
Smilowitz 2014 [12]	rPCI	40	PCS	64.4 ± 9.5	13/27	NR	35(87.5)	24(60)	16(40.0)	13.7 ± 4.8	NR
	mPCI	80		67.4 ± 10.5	27/53		74(92.5)	48(60)	35(43.8)	13.9 ± 5.9	
Muhlen 2025 [19]	rPCI	70	PCS	69.8 ± 12.6	16/54	28.0 ± 4.8	50(71.4)	32(45.7)	30(42.9)	NR	51(72.9)
	mPCI	70		77.1 ± 10.4	14/56	26.0 ± 4.5	51(72.9)	19(27.1)	25(35.7)		50(71.4)
Patel 2020 [17]	rPCI	310	RCS	58.9 ± 10.4	60/250	NR	229(73.9)	29(9.4)	181(58.4)	NR	NR
	mPCI	686		58.1 ± 11.1	167/519		404(58.9)	50(7.3)	290(42.3)		
Walters 2018 [11]	rPCI	108	PCS	68.1 ± 11.0	22/86	NR	98(95.4)	NR	58(56.0)	22.2 ± 10.6	13(12.0)
	mPCI	210		67.5 ± 12.1	46/164		199(95.10)		113(54.0)	19.4 ± 9.5	28(12.4)
Yu 2025 [14]	rPCI	73	RCT	64.2 ± 9.6	25/48	21.35 ± 2.80	55(75.3)	26(35.6)	27(37.0)	18.1 ± 5.2	72(98.6)
	mPCI	79		61.1 ± 11.1	25/54	21.48 ± 2.77	51(64.6)	27(34.2)	26(32.9)	20.1 ± 8.9	77(97.5)

*rPCI* Robotic-assisted percutaneous coronary intervention, *mPCI* Manual percutaneous coronary intervention, *RCT* Randomized controlled trial, *RCS* Retrospective cohort study, *PCS* Prospective cohort study, *NR* Not reported, *BMI* Body mass index

**Table 2:** Results from 11 studies

Study ID (Year)	Group	Cases (*n*)	Study design	Technical Success Rate *n* (%)	Clinical Success Rate *n* (%)		Procedural duration (min)	TIMI 3 flow after PCI *n* (%)	MACE *n* (%)	Coronary Dissection after PCI *n* (%)	Fluoroscopy time (min)	Contrast volume (mL)	DAP (dGy·cm²)	RAK (mGy)	Operator radiation exposure (µSv)	12-month all-cause mortality *n* (%)
Bay 2024 [18]	rPCI	85	PCS	85(100.0)	NR		NR	85(100)	3(3.52)	1(1.1)	NR	146.80 ± 62.22	230.9 ± 192.5	NR	NR	2(2.35)
	mPCI	226		213(94.2)		NR		218(96.5)	15(6.5)	3(1.3)		144.25 ± 67.78	213.5 ± 185.4			11(4.86)
Hirai 2020 [34]	rPCI	49	RCS	48(98.0)	NR		89.6 ± 27.1	NR	3(6.12)	2(4.08)	37.9 ± 17.9	111 ± 39	NR	1522 ± 1129	NR	NR
	mPCI	46		46(100.0)			93.4 ± 30.5		6(13.0)	2(4.34)	48.6 ± 17.1	118 ± 53		1966 ± 1204		
Kagiyama 2021 [9]	rPCI	30	RCS	27(90.0)	28(93.3)		72.4 ± 41.2	NR	0(0)	NR	27.5 ± 18.9	93.2 ± 44.5	275.0 ± 112.3	NR	0.96 ± 0.3	NR
	mPCI	77		71(92.2)	71(92.2)		65.6 ± 34.8		0(0)		30.1 ± 14.9	102.0 ± 32.9	324.5 ± 143.2		26.2 ± 19.5	
Madder 2016 [10]	rPCI	45	PCS	43(95.55)	NR		55.1 ± 11.9	NR	NR	NR	11.70 ± 7.26	167.00 ± 115.5	385.6 ± 136.5	1272.12 ± 302.22	0.25 ± 0.12	NR
	mPCI	168		164(97.6)			48.2 ± 23.0				12.80 ± 6.52	161.00 ± 96.30	455.6 ± 153.4	1507.20 ± 275.56	38.1 ± 30.8	
Mahmud 2017 [13]	rPCI	108	RCS	103(95.6)	107(99.1)		44.30 ± 26.04	NR	1(0.92)	2(1.9)	22.3 ± 10.4	183.4 ± 78.7	251.8 ± 157.0	NR	0.75 ± 0.25	1(0.92)
	mPCI	226		207(91.7)	220(97.34)		36.34 ± 23.03		6(2.65)	3(1.3)	28.6 ± 11.4	202.5 ± 74.0	404.8 ± 143.7		19.88 ± 10.65	6(2.65)
Mangels 2020 [15]	rPCI	56	RCS	NR	56(100.0)		NR	NR	1(1.78)	NR	NR	184.8 ± 39.4	NR	NR	NR	NR
	mPCI	108			105(97.2)				4(3.7)			217.9 ± 37.8				
Smilowitz 2014 [12]	rPCI	40	PCS	38(95.0)	39(97.5)		NR	39(97.5)	NR	NR	10.1 ± 4.7	121 ± 47	138.9 ± 59.9	NR	1.15 ± 1.05	NR
	mPCI	80		74(92.5)	76(95.0)			76(95)			19.8 ± 7.6	137 ± 62	166.5 ± 102.6		26.30 ± 18.66	
Muhlen 2025 [19]	rPCI	70	PCS	70(100.0)	70(100.0)		103.1 ± 22.2	70(100)	0(0.0)	0(0.0)	14.5 ± 8.52	180.25 ± 81.48	406.1 ± 270.2	NR	NR	0(0)
	mPCI	70		66(94.36)	69(98.6)		67.3 ± 20.7	66(94.3)	3(4.28)	1(1.42)	18.2 ± 8.15	160.00 ± 51.85	324.1 ± 201.7			3(4.28)
Patel 2020 [17]	rPCI	310	RCS	NR	NR		NR	NR	NR	NR	15.88 ± 4.36	140.56 ± 59.26	473.4 ± 374.1	884.50 ± 637.78	NR	6(1.93)
	mPCI	686									26.75 ± 4.04	136.50 ± 49.63	574.6 ± 302.3	1110.45 ± 591.85		22(3.20)
Walters 2018 [11]	rPCI	108	PCS	99(91.7)	107(99.07)		NR	NR	3(2.77)	2(1.85)	NR	NR	NR	NR	NR	3(2.77)
	mPCI	210		200(95.2)	201(95.71)				7(3.33)	3(1.42)						9(4.28)
Yu 2025 [14]	rPCI	73	RCT	73(100.0)	73(100.0)		60.1 ± 17.0	73(100)	NR	NR	11 ± 5.93	90.00 ± 51.85	NR	836.75 ± 979.63	0.15 ± 0.1	NR
	mPCI	79		79(100.0)	79(100.0)		53.1 ± 17.8	78(98.9)			15 ± 7.41	81.67 ± 37.04		796.5 ± 937.22	54.08 ± 73.60	

*PCI* Percutaneous coronary intervention, *TIMI* Thrombolysis in myocardial infarction, *n* Number, *min* Minutes, *MACE* Major adverse cardiovascular events, *DAP* Dose-area product, *RAK* Radiation air Kerma, *NR* Not reported

**Fig. 2:**
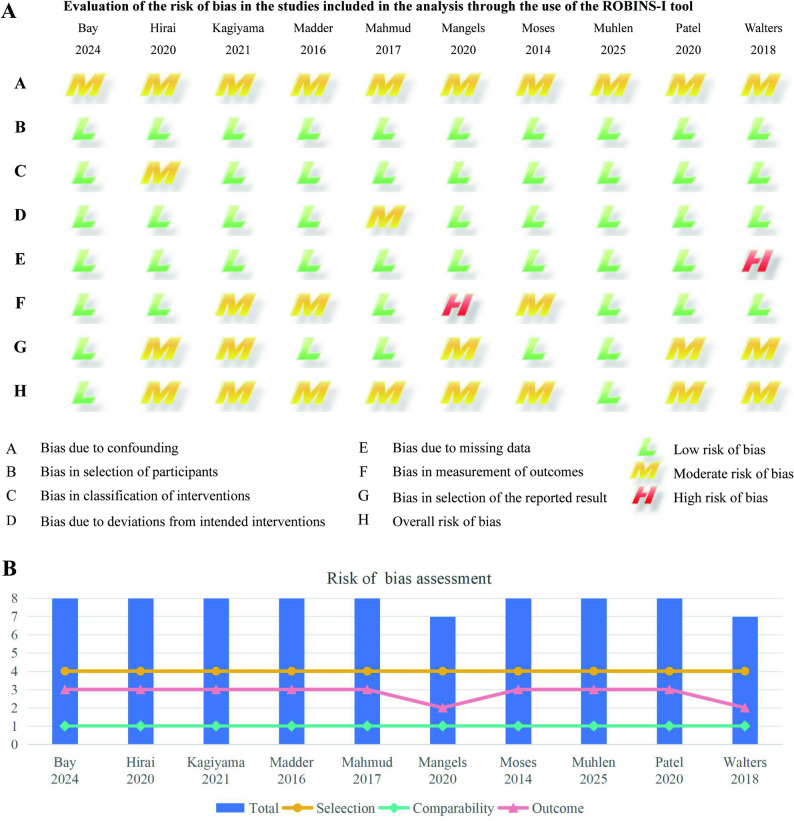
**A** ROBINS-I risk-of-bias assessment for the included non-randomized studies. **B** Newcastle–Ottawa Scale–based methodological quality assessment of the included studies

**Fig. 3:**
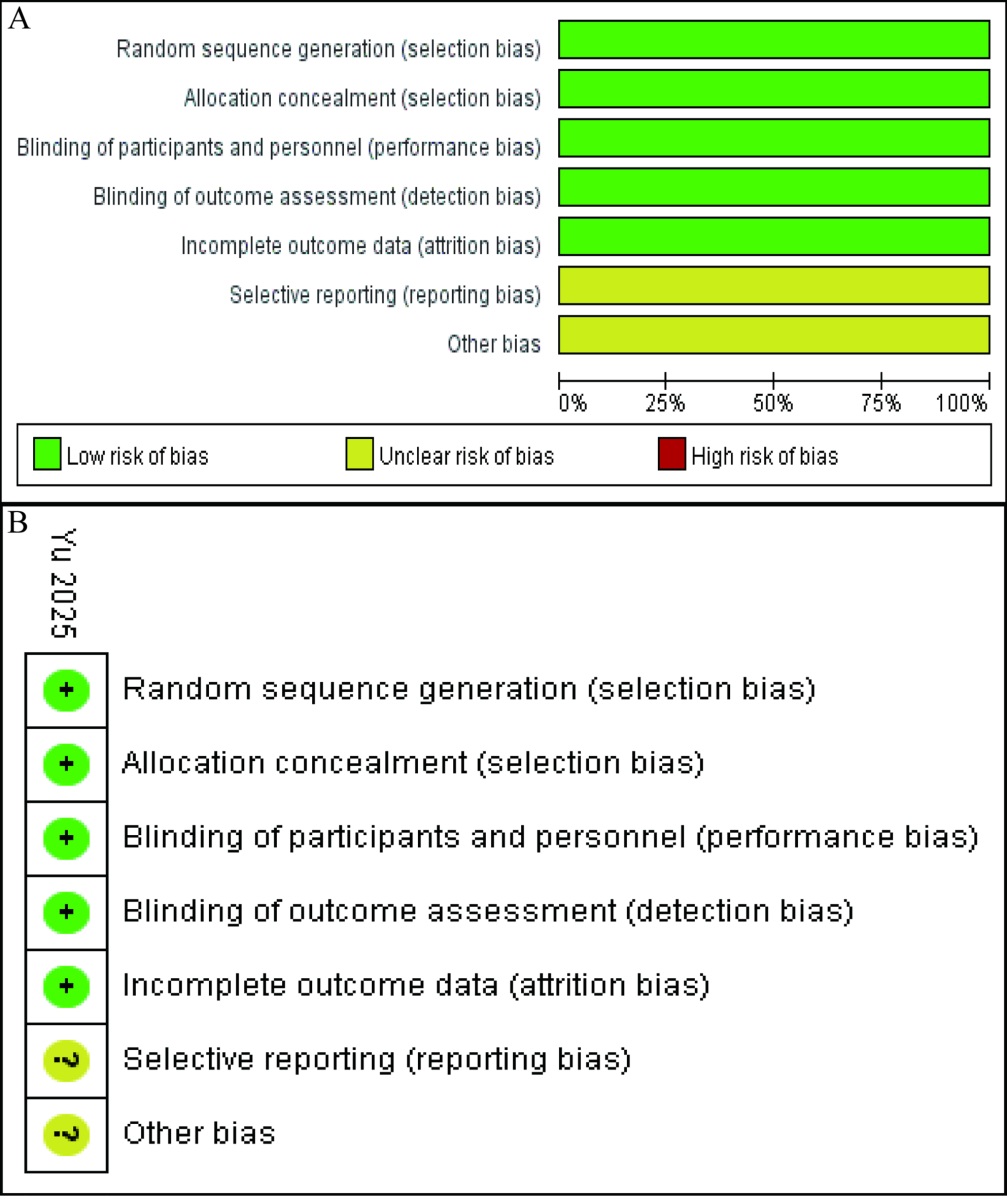
Risk-of-bias assessment of the included randomized controlled trial using the Cochrane tool. Red, high risk; yellow, unclear risk; green, low riskRisk-of-bias assessment of the included randomized controlled trial using the Cochrane tool. **A** Risk-of-bias summary plot showing the percentage of studies at low, unclear, and high risk of bias across each domain. **B** Risk-of-bias assessment for the individual included study (Yu 2025). Red, high risk; yellow, unclear risk; green, low risk

A study-level risk-of-bias assessment using the ROBINS-I tool is shown in Fig. 2A. Overall, most included studies were judged to be at moderate risk of bias, although two studies were assessed as having low overall risk. Study quality assessment is shown in Fig. 2B. Overall, most included studies were of relatively high methodological quality, although some limitations were noted in specific domains. The single randomized controlled trial was assessed using the Cochrane risk-of-bias tool and was judged to be of good methodological quality (Figs. 3A–B).

### Fluoroscopy time

**Fig. 4:**
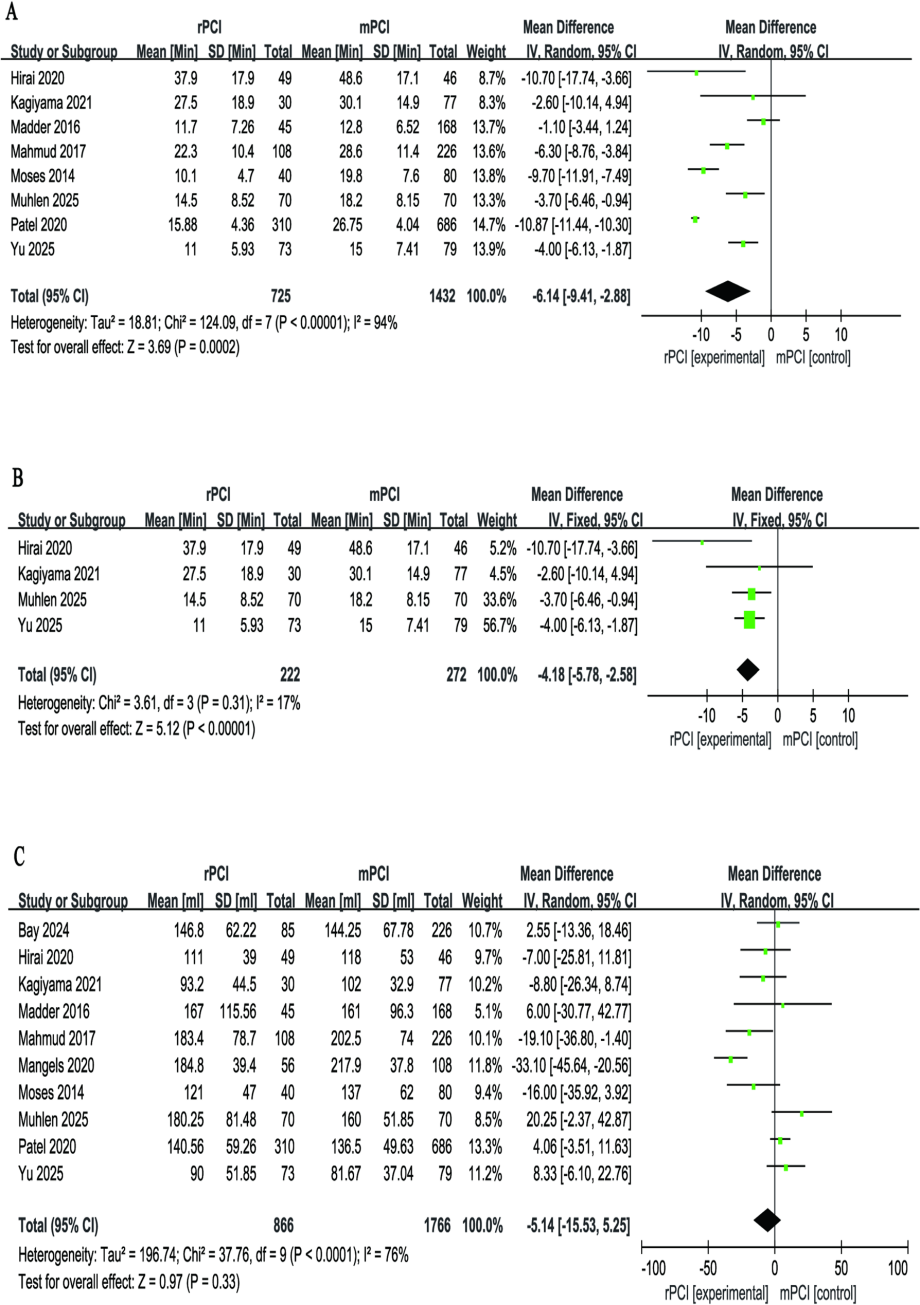
**A**-**B**: Fluoroscopy time (min) and its subgroup analysis; **C**: contrast volume (mL)

A total of 8 studies involving 2157 patients (rPCI: 725; mPCI: 1432) were included in this analysis [9, 10, 12–14, 17, 19, 34]. Substantial heterogeneity was observed across the included studies (I² = 94%), and therefore a random-effects model was applied. In the pooled analysis, fluoroscopy time was shorter in the rPCI group than in the mPCI group (MD = − 6.14 min, 95% CI: −9.41 to − 2.88, *P* = 0.0002; Fig. 4A).

Because heterogeneity was marked, an exploratory post hoc subgroup analysis was performed including only studies published in or after 2020, in order to reflect more contemporary robotic platforms and procedural practice. This subgroup comprised 4 studies involving 494 patients (rPCI: 222; mPCI: 272) [9, 14, 19, 34]. In this subgroup, heterogeneity was substantially reduced (I² = 17%), and a fixed-effects model was applied. The direction of effect remained unchanged, with shorter fluoroscopy time still observed in the rPCI group (MD = − 4.18 min, 95% CI: −5.78 to − 2.58; *P* < 0.00001; Fig. 4B). These findings suggest that temporal differences in study period and procedural maturation may have contributed to the heterogeneity of the overall analysis.

## Contrast volume

A total of 10 studies involving 2632 patients (rPCI: 866; mPCI: 1766) were included in this analysis [9, 10, 12–15, 17–19, 34]. This outcome reflects the total volume of contrast medium used during PCI procedures. Moderate to substantial heterogeneity was observed across the included studies (I² = 76%), and therefore a random-effects model was applied. Contrast volume was comparable between groups (MD = − 5.14 mL, 95% CI: −15.53 to 5.25; *P* = 0.33; Fig. 4C).

## Dose-area product

**Fig. 5:**
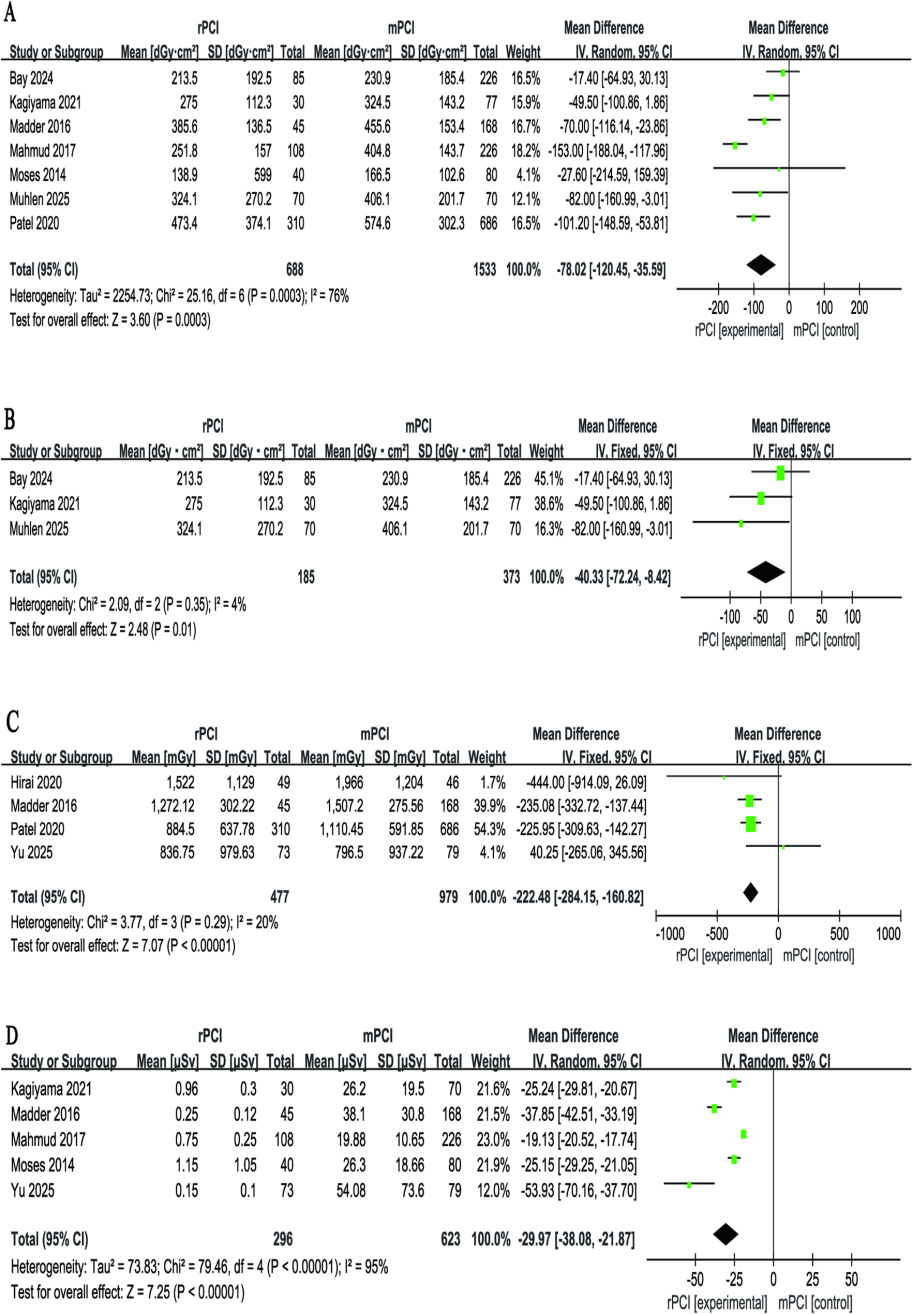
**A**-**B**: Dose-area product (DAP, dGy·cm²) and its subgroup analysis; **C**: radiation air kerma (RAK, mGy); **D**: operator radiation exposure (µSv)

A total of 7 studies involving 2221 patients (rPCI: 688; mPCI: 1533) were included in this analysis [9, 10, 12, 13, 17–19]. This outcome reflects the dose–area product during PCI procedures. Moderate to substantial heterogeneity was observed across the included studies (I² = 76%), and therefore a random-effects model was applied. The pooled analysis showed that the dose–area product was significantly lower in the rPCI group compared with the mPCI group (MD = − 78.02 dGy·cm², 95% CI: −120.45 to − 35.59, *P* = 0.0003; Fig. 5A).

To further investigate whether temporal differences in study period and evolving procedural practice contributed to heterogeneity, an exploratory post hoc subgroup analysis was conducted including only studies published in or after 2020. This subgroup comprised 3 studies involving 558 patients (rPCI: 185; mPCI: 373) [9, 18, 19]. In this subgroup, heterogeneity was markedly reduced (I²=4%), and a fixed-effects model was applied. The results remained consistent, demonstrating a significantly lower dose–area product in the rPCI group compared with the mPCI group (MD = -40.33 dGy·cm², 95% CI: -72.24 to -8.42, *P* = 0.01; Fig. 5B).

### Radiation air Kerma

A total of 4 studies involving 1456 patients (rPCI: 477; mPCI: 979) were included in this analysis [10, 14, 17, 34]. This outcome reflects the air kerma during PCI procedures. Low heterogeneity was observed across the included studies (I²=20%), and therefore a fixed-effects model was applied. Air kerma was lower in the rPCI group (MD = − 222.48 mGy, 95% CI: −284.15 to − 160.82, *P* < 0.00001; Fig. 5C).

### Operator radiation exposure

A total of 5 studies involving 919 patients (rPCI: 296; mPCI: 623) were included in this analysis [9, 10, 12–14]. This outcome, measured in microsieverts (µSv), reflects the ionizing radiation dose received by the operator during PCI procedures and serves as a key indicator of occupational radiation exposure. Substantial heterogeneity was observed across the included studies (I² = 95%), and therefore a random-effects model was applied. The pooled analysis showed that operator radiation exposure was lower in the rPCI group compared with the mPCI group (MD = − 29.97 µSv, 95% CI: −38.08 to − 21.87, *P* < 0.00001; Fig. 5D).

### Procedural duration

**Fig. 6:**
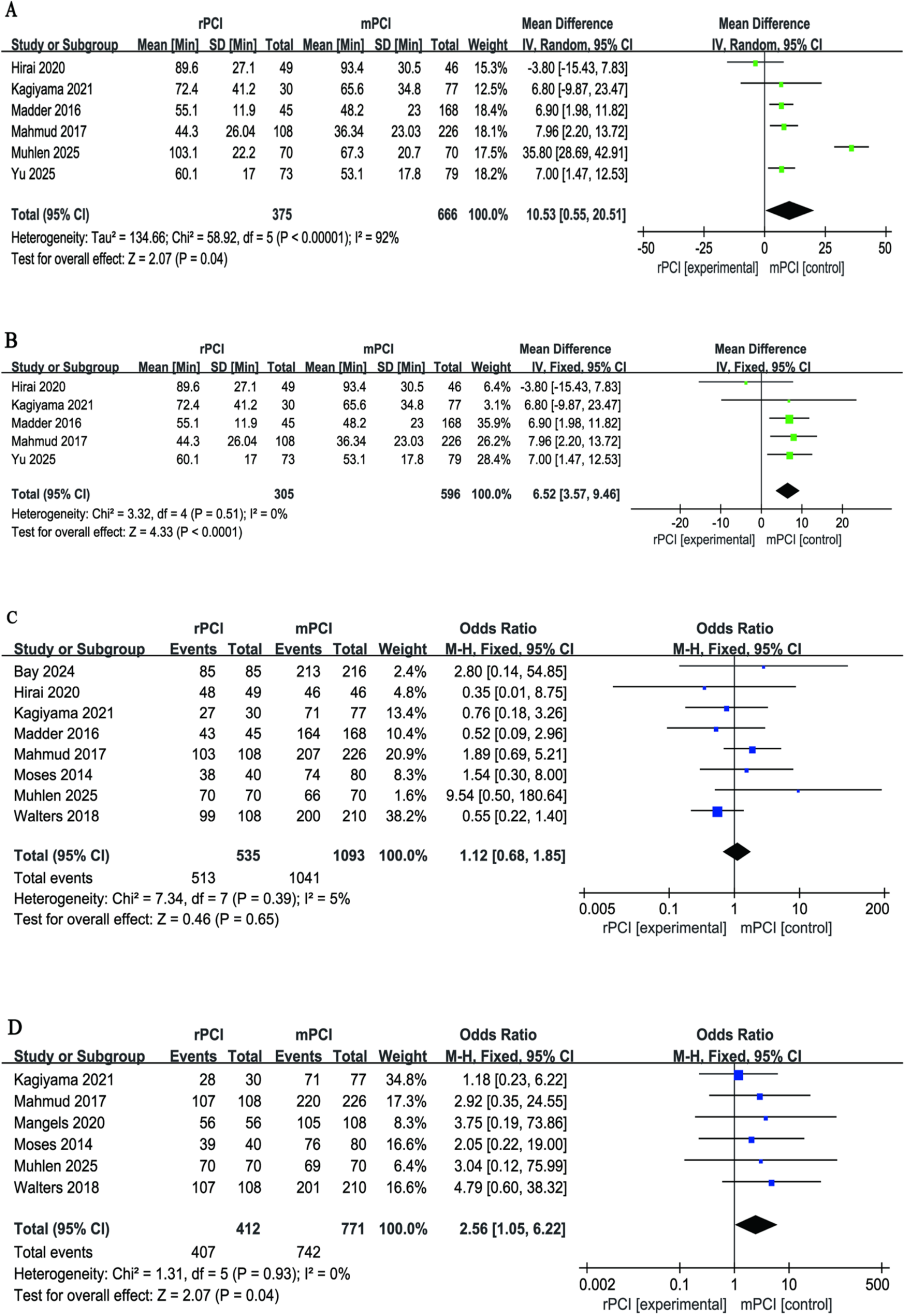
**A**-**B**: Procedural duration (min) and its subgroup analysis; **C**: technical success; **D**: clinical success

A total of 6 studies involving 1041 patients (rPCI: 375; mPCI: 666) were included in this analysis [9, 10, 13, 14, 19, 34]. This outcome reflects the total procedural duration during PCI. Substantial heterogeneity was observed across the included studies (I² = 92%), and therefore a random-effects model was applied. In the pooled analysis, procedural duration was longer in the rPCI group than in the mPCI group (MD = 10.53 min, 95% CI: 0.55–20.51; *P* = 0.04; Fig. 6A).

Because heterogeneity was marked, a post hoc leave-one-out sensitivity analysis was undertaken to explore the influence of individual studies on the pooled estimate. This analysis identified the von Zur Mühlen 2025 study as a major contributor to between-study heterogeneity. After exclusion of this study, 5 studies involving 901 patients (rPCI: 305; mPCI: 596) remained [9, 10, 13, 14, 34], and heterogeneity was eliminated (I² = 0%). The pooled effect remained directionally consistent, with longer procedural duration still observed in the rPCI group (MD = 6.52 min, 95% CI: 3.57 to 9.46; *P* < 0.0001; Fig. 6B). These findings suggest that the association between rPCI and longer procedural duration was robust, while also indicating that differences in study characteristics contributed materially to the heterogeneity of the overall analysis.

### Technical success

A total of 8 studies involving 1628 patients (rPCI: 535; mPCI: 1093) were included in this analysis, with 513 and 1041 successful procedures in the rPCI and mPCI groups, respectively [9–13, 18, 19, 34]. Low heterogeneity was observed across the included studies (I² = 5%), and therefore a fixed-effects model was applied. Technical success was similar between groups (OR = 1.12, 95% CI: 0.68–1.85; *P* = 0.65; Fig. 6C).

### Clinical success

A total of 6 studies involving 1,183 patients (rPCI: 412; mPCI: 771) were included in this analysis, with 407 and 742 successful outcomes in the rPCI and mPCI groups, respectively [9, 11–13, 15, 19]. The pooled analysis showed higher clinical success in the rPCI group (OR = 2.56, 95% CI: 1.05–6.22; *P* = 0.04). Notably, while definitions of clinical success varied among the included studies, they generally included achievement of procedural objectives such as successful stent deployment at the target lesion, residual stenosis below a defined threshold, restoration of post-procedural TIMI grade 3 flow, and no requirement for repeat revascularization during hospitalization. Despite these definitional differences, the observed heterogeneity was 0% (I² = 0%), indicating that these inter-study variations did not materially affect the pooled estimate.

### Major adverse cardiovascular events

**Fig. 7:**
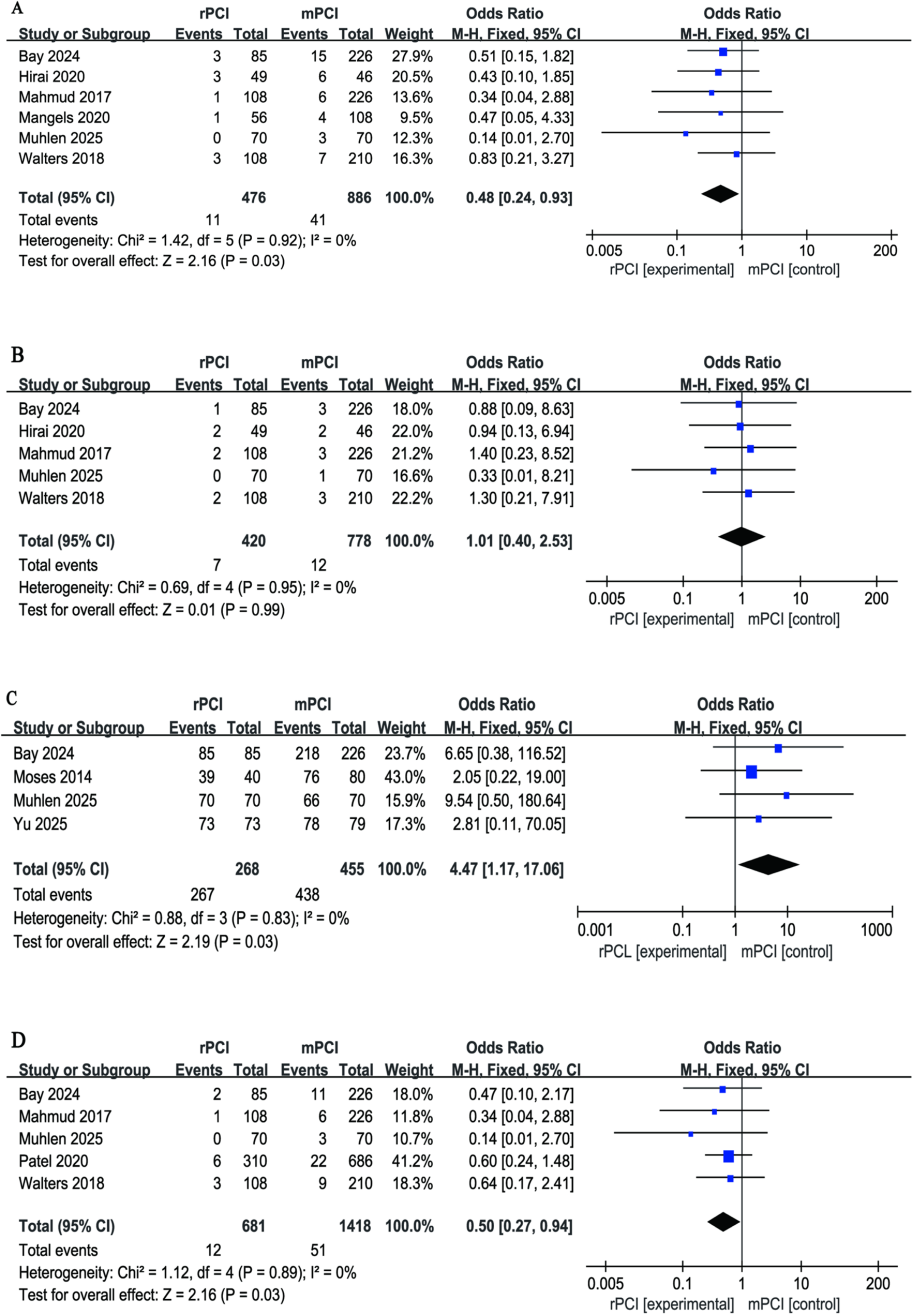
**A**: major adverse cardiovascular events (MACE); **B**: Coronary Dissection; **C**: TIMI 3 flow restoration; **D**: All-cause mortality at 12-month follow-up

A total of 6 studies involving 1362 patients (rPCI: 476; mPCI: 886) were included in this analysis, with 11 and 41 MACE reported in the rPCI and mPCI groups [11, 13, 15, 18, 19, 34]. This outcome reflects the incidence of adverse cardiovascular events following PCI, typically including death, myocardial infarction, and target vessel revascularization. No heterogeneity was observed across the included studies (I² = 0%), and therefore a fixed-effects model was applied. The pooled analysis showed that the incidence of MACE was lower in the rPCI group compared with the mPCI group (OR = 0.48, 95% CI: 0.24 to 0.93, *P* = 0.03; Fig. 7A). The specific components of MACE in each included study are summarized in Supplementary Table 1.

### Postprocedural coronary dissection

A total of 5 studies involving 1198 patients (rPCI: 420; mPCI: 778) were included in this analysis, with 7 and 12 arterial dissections reported in the rPCI and mPCI groups [11, 13, 18, 19, 34]. No heterogeneity was observed across the included studies (I² = 0%), and therefore a fixed-effects model was applied. Postprocedural coronary dissection rates were comparable between the rPCI and mPCI groups (OR = 1.01, 95% CI: 0.40–2.53; *P* = 0.99; Fig. 7B).

### Postprocedural TIMI 3 flow restoration

A total of 4 studies involving 723 patients (rPCI: 268; mPCI: 455) were included in this analysis, with 267 and 438 patients achieving TIMI grade 3 flow in the rPCI and mPCI groups [12, 14, 18, 19]. This outcome reflects the restoration of optimal coronary blood flow following PCI. No heterogeneity was observed across the included studies (I² = 0%), and therefore a fixed-effects model was applied. The pooled analysis showed that the rate of TIMI grade 3 flow was significantly higher in the rPCI group compared with the mPCI group (OR = 4.47, 95% CI: 1.17 to 17.06, *P* = 0.03; Fig. 7C).

### All-cause mortality at 12-month follow-up

A total of 5 studies involving 2099 patients (rPCI: 681; mPCI: 1418) were included in this analysis, with 12 and 51 deaths reported in the rPCI and mPCI groups [11, 13, 17–19]. This outcome reflects all-cause mortality at 12 months following PCI. No heterogeneity was observed across the included studies (I² = 0%), and therefore a fixed-effects model was applied. The pooled analysis showed that 12-month all-cause mortality was significantly lower in the rPCI group compared with the mPCI group (OR = 0.50, 95% CI: 0.27 to 0.94, *P* = 0.03; Fig. 7D).

## Discussion

In this systematic review and meta-analysis of 11 comparative studies including 2,950 patients, the most reproducible advantage of robotic-assisted PCI was a reduction in radiation-related burden, particularly for the operator. By contrast, procedural efficiency appeared less favorable, as rPCI was associated with longer procedural duration. Signals for improved procedural and follow-up outcomes were also observed, but these findings should be interpreted more cautiously because they were derived predominantly from non-randomized studies and relatively limited event counts. Overall, the current evidence supports rPCI primarily as a strategy that improves occupational protection without compromising procedural safety, while its incremental benefit for patient-level clinical outcomes remains to be confirmed. Compared with the prior meta-analysis by Tripathi et al. (2021) [23], which included 5 studies with 1,555 patients, the present study incorporates a larger number of studies and patients and evaluates additional clinically relevant outcomes, including MACE, 12-month all-cause mortality, postprocedural TIMI 3 flow, postprocedural coronary dissection, and technical success rate. Furthermore, radiation safety was assessed using more standardized and quantitative metrics, including DAP, RAK, and operator radiation exposure, and studies published after 2021 were included. With these improvements, we summarize the latest procedural and patient-level efficacy and safety profiles of rPCI versus mPCI.

The clearest and most robust radiation-related finding of the present analysis was the reduction in operator radiation exposure, which represents the most direct measure of occupational protection. Compared with manual PCI, robotic-assisted PCI was associated with lower operator radiation exposure, supporting the primary rationale for robotic PCI as a strategy to reduce cumulative radiation burden and ergonomic risk for interventional operators [10, 35]. By contrast, patient- and procedure-related radiation metrics, including dose-area product, air kerma, and fluoroscopy time, should be interpreted separately from operator exposure. Although pooled estimates favored rPCI for these endpoints, reductions in patient radiation burden were less consistent across individual studies and are more susceptible to case complexity, imaging settings, laboratory workflow, robotic platform generation, and operator experience. Therefore, the principal radiation-related conclusion of this meta-analysis is that rPCI provides a consistent occupational safety advantage, whereas potential reductions in patient radiation exposure should be considered supportive but less definitive. The marked reduction in heterogeneity in exploratory subgroup analyses restricted to more contemporary studies, together with preservation of the direction of effect, suggests that part of the between-study variability may reflect platform maturation and workflow standardization. This distinction is important in interventional cardiology, where cumulative operator radiation exposure and the orthopedic burden of lead protection remain persistent occupational concerns.

The procedural trade-off was less favorable. Although contrast use was similar between groups, rPCI was associated with longer procedural duration. This pattern suggests that the time penalty of robotic PCI is more likely related to setup, device exchange, workflow adaptation, and learning curve than to ineffective lesion treatment itself. The absence of an increase in contrast volume is relevant here, because it argues against a broader loss of procedural control. In practical terms, rPCI appears to introduce workflow cost without clear evidence of greater intraprocedural resource burden [8, 10]. The reduction in heterogeneity after exclusion of an influential study further suggests that the observed prolongation in procedural duration is not merely a statistical artifact, but a reproducible feature of current robotic workflows. Viewed together, the combination of longer procedural duration, unchanged contrast use, and preserved technical success suggests that the main trade-off of rPCI lies in workflow complexity rather than loss of procedural efficacy.

The procedural and angiographic findings warrant interpretation as a group rather than as isolated endpoints. Technical success was similar between rPCI and mPCI, with no increase in postprocedural coronary dissection, indicating that robotic assistance does not compromise procedural feasibility or mechanical safety. Clinical success and postprocedural TIMI 3 flow appeared higher with rPCI (OR = 2.56 and OR = 4.47, respectively); however, these effect sizes should be interpreted with caution. The findings are derived from a small number of studies with limited sample sizes, predominantly non-randomized observational data. Potential biases, including selection bias, residual confounding, and differences in lesion complexity and operator experience, may contribute to the observed magnitudes. Mechanistically, robotic systems may allow more controlled device movement and more objective lesion-length assessment, which could support more precise stent deployment in selected lesions, but these explanations remain speculative. Consequently, while these results suggest a possible procedural benefit of rPCI, they may not fully reflect the true biological or clinical effect, and further adequately powered randomized trials are needed to validate these procedural signals. In addition, the estimate for TIMI 3 flow was based on a relatively small number of studies and should not be overinterpreted.

The follow-up findings related to MACE and 12-month all-cause mortality should be interpreted cautiously. Although rPCI was associated with lower rates of these outcomes, the evidence is primarily derived from observational studies with limited events. These findings should be considered hypothesis-generating, not causal. Patient-level outcomes may be influenced by factors such as lesion complexity, operator experience, and center-level variations. Furthermore, patient selection by highly experienced operators, often in high-volume centers, may independently contribute to these favorable clinical outcomes. Operators experience also plays a crucial role in managing complex lesions and optimizing procedural techniques, which may affect the rates of MACE and mortality observed in the rPCI group. While these results suggest potential benefits of rPCI, they should be interpreted with caution due to the limitations in the study design. Future research should focus on large-scale, well-designed RCT that can isolate the effects of the robotic platform from confounding factors such as operator expertise and patient selection. Additionally, RCT should explore the long-term clinical outcomes of rPCI, especially in complex coronary lesions and diverse patient populations.

From a clinical standpoint, the present findings suggest that the strongest current rationale for adopting rPCI is operator protection rather than proven superiority in hard patient outcomes. That distinction matters. A technology may have legitimate value in contemporary catheterization laboratories if it reduces cumulative occupational harm while preserving procedural success and safety. At the same time, the longer procedural duration observed in this analysis indicates that wider implementation of rPCI should be accompanied by careful workflow integration, training, and case selection. In this sense, the present evidence supports rPCI as a safe and ergonomically favorable adjunct to PCI, with potential procedural and clinical advantages that remain promising rather than definitive.

### Limitations and future research

Several limitations should be considered. First, most included studies were observational, leaving room for residual confounding and center-level selection effects. Second, substantial heterogeneity was observed for several key endpoints, including fluoroscopy time, operator radiation exposure, and procedural duration. While subgroup and sensitivity analyses were performed, the sources of heterogeneity may include temporal trends, differences in robotic platforms, operator experience, and study design. Third, differences in lesion complexity and procedural characteristics across studies may have substantially influenced both procedural and clinical outcomes. Key variables, including SYNTAX score, lesion type (e.g., bifurcation, calcified lesions, chronic total occlusions), and procedural approach, were inconsistently reported or absent in many studies. As a result, our pooled estimates could not fully adjust for these factors, and residual confounding from unmeasured lesion- or procedure-specific variables should be acknowledged. Fourth, some clinically relevant endpoints, particularly TIMI 3 flow and 12-month mortality, were based on relatively few studies or events. Fifth, definitions of composite outcomes such as MACE were not fully uniform across studies. Sixth, formal assessment of publication bias was limited because most outcomes were informed by fewer than 10 studies. Therefore, larger, randomized controlled trials are needed to confirm these findings and better separate the effects of the robotic system from those of operator expertise and patient selection. These trials will help clarify the broader clinical applicability of rPCI, including its procedural efficiency, resource utilization, learning-curve effects, and economic implications.

## Conclusions

Current comparative evidence suggests that rPCI may offer an occupational safety advantage, particularly in reducing operator radiation exposure, while preserving procedural safety compared to mPCI. However, the potential reductions in patient- or procedure-related radiation metrics are not consistently observed, and these findings should be interpreted with caution. The available data also suggest possible gains in selected procedural and clinical outcomes, but these signals remain less secure than the radiation-related benefit and are derived largely from non-randomized evidence. At present, rPCI should be viewed primarily as a technology with a clear ergonomic and radiation-related rationale, while its incremental benefit for long-term patient outcomes requires confirmation in larger, adequately powered randomized studies.

## Supplementary information

Supplementary material 1.

Supplementary material 2.

Supplementary material 3.

Supplementary material 4.

## Data Availability

The datasets used and/or analysed during the current study are available from the corresponding author on reasonable request.
