# Supplementary material for: Operator radiation burden and periprocedural outcomes in robotic-assisted versus manual percutaneous coronary intervention: a meta-analysis
Source: BMC Surg. 2026 May 22;26:476. doi: 10.1186/s12893-026-03863-7 (PMC13371123; doi:10.1186/s12893-026-03863-7)
Supplement: Supplementary file 2 — Supplementary material 2. [file 12893_2026_3863_MOESM2_ESM.docx]

**Supplementary Table 1: Definition of Major Adverse Cardiovascular Events (MACE) in Included Studies**

| **Study ID (Year)** | **Sample Size (rPCI / mPCI)** | **MACE Definition** | **Components** |
| --- | --- | --- | --- |
| Hirai 2020 | 49/46 | Composite adverse clinical events | Death, MI, clinical perforation, significant vessel dissection, arrhythmia, acute thrombus, stroke |
| Kagiyama 2021 | 30/77 | Cardiac death, MI, clinically driven target vessel revascularization | Cardiac death, MI, TVR |
| Mahmud 2017 | 108/226 | In-hospital major adverse cardiovascular events | Death, stroke, emergent CABG, target vessel revascularization, nonfatal MI |
| Bay 2024 | 85/226 | Adverse clinical event | Cardiac or non-cardiac death, ischemic or hemorrhagic stroke, access-site complication, major or minor TIMI bleeding, urgent target-vessel revascularization, MI |
| Muhlen 2025 | 70/70 | Composite MACE | Death, stroke, MI, repeat PCI or CABG |
| Walters 2018 | 103/210 | Major adverse cardiovascular events | Death, stroke, MI, TVR |

**Abbreviations:** rPCI: Robotic-assisted percutaneous coronary intervention; mPCI: Manual percutaneous coronary intervention; MI: Myocardial infarction; TVR: Target vessel revascularization; CABG: Coronary artery bypass grafting; TIMI: Thrombolysis in Myocardial Infarction scale
