# Supplementary material for: Operator radiation burden and periprocedural outcomes in robotic-assisted versus manual percutaneous coronary intervention: a meta-analysis
Source: BMC Surg. 2026 May 22;26:476. doi: 10.1186/s12893-026-03863-7 (PMC13371123; doi:10.1186/s12893-026-03863-7)
Supplement: Supplementary file 4 — Supplementary material 4. [file 12893_2026_3863_MOESM4_ESM.docx]

| **Section and Topic** | **Item #** | **Checklist item** | **Location where item is reported** |  |
| --- | --- | --- | --- | --- |
| **TITLE** | | |  |  |
| Title | 1 | Identify the report as a systematic review. | Page 1, lines 2-3 |  |
| **ABSTRACT** | | |  |  |
| Abstract | 2 | See the PRISMA 2020 for Abstracts checklist. | Page 2, lines 25-51 |  |
| **INTRODUCTION** | | |  |  |
| Rationale | 3 | Describe the rationale for the review in the context of existing knowledge. | Page 3, Introduction, paragraphs 1–3 |  |
| Objectives | 4 | Provide an explicit statement of the objective(s) or question(s) the review addresses. | Page 3, Introduction, final paragraph |  |
| **METHODS** | | |  |  |
| Eligibility criteria | 5 | Specify the inclusion and exclusion criteria for the review and how studies were grouped for the syntheses. | Page 4, Methods, Inclusion Criteria and Data Extraction, paragraphs 1–2. |  |
| Information sources | 6 | Specify all databases, registers, websites, organisations, reference lists and other sources searched or consulted to identify studies. Specify the date when each source was last searched or consulted. | Page 4-5, Methods, Literature Search Strategy, paragraph 1. |  |
| Search strategy | 7 | Present the full search strategies for all databases, registers and websites, including any filters and limits used. | Page 4-5, Methods, Literature Search Strategy, paragraph 1 |  |
| Selection process | 8 | Specify the methods used to decide whether a study met the inclusion criteria of the review, including how many reviewers screened each record and each report retrieved, whether they worked independently, and if applicable, details of automation tools used in the process. | Page 4-5, Methods, Literature Search Strategy, paragraph 1. |  |
| Data collection process | 9 | Specify the methods used to collect data from reports, including how many reviewers collected data from each report, whether they worked independently, any processes for obtaining or confirming data from study investigators, and if applicable, details of automation tools used in the process. | Page 4-5, Methods, Literature Search Strategy, paragraph 1; Page 4-5, Methods, Inclusion Criteria and Data Extraction, paragraph 1. |  |
| Data items | 10a | List and define all outcomes for which data were sought. Specify whether all results that were compatible with each outcome domain in each study were sought (e.g. for all measures, time points, analyses), and if not, the methods used to decide which results to collect. | Page 4-5, Methods, Inclusion Criteria and Data Extraction, paragraph 2. |  |
|  | 10b | List and define all other variables for which data were sought (e.g. participant and intervention characteristics, funding sources). Describe any assumptions made about any missing or unclear information. | Page 4-5, Methods, Inclusion Criteria and Data Extraction, paragraph 1 |  |
| Study risk of bias assessment | 11 | Specify the methods used to assess risk of bias in the included studies, including details of the tool(s) used, how many reviewers assessed each study and whether they worked independently, and if applicable, details of automation tools used in the process. | Page 6-7, Methods, Quality Assessment, Publication Bias, and Statistical Analysis. |  |
| Effect measures | 12 | Specify for each outcome the effect measure(s) (e.g. risk ratio, mean difference) used in the synthesis or presentation of results. | Page 6-7, Methods, Quality Assessment, Publication Bias, and Statistical Analysis. |  |
| Synthesis methods | 13a | Describe the processes used to decide which studies were eligible for each synthesis (e.g. tabulating the study intervention characteristics and comparing against the planned groups for each synthesis (item #5)). | Page 4, Methods, Inclusion Criteria and Data Extraction, where prespecified outcomes and study grouping are described; Page 5–8, Results, where eligible studies for each outcome-specific synthesis are reported. |  |
|  | 13b | Describe any methods required to prepare the data for presentation or synthesis, such as handling of missing summary statistics, or data conversions. | Page 4-7, Methods, Quality Assessment, Publication Bias, and Statistical Analysis, where conversion of medians and interquartile ranges to means and standard deviations is described. |  |
|  | 13c | Describe any methods used to tabulate or visually display results of individual studies and syntheses. | Page 6-7, Methods, Quality Assessment, Publication Bias, and Statistical Analysis; Page 7–12, Results, Study Selection and Characteristics and outcome-specific synthesis sections; Tables 1–2 and Figures 1–7. |  |
|  | 13d | Describe any methods used to synthesize results and provide a rationale for the choice(s). If meta-analysis was performed, describe the model(s), method(s) to identify the presence and extent of statistical heterogeneity, and software package(s) used. | Page 6-7, Methods, Quality Assessment, Publication Bias, and Statistical Analysis. |  |
|  | 13e | Describe any methods used to explore possible causes of heterogeneity among study results (e.g. subgroup analysis, meta-regression). | Page 6-7, Methods, Quality Assessment, Publication Bias, and Statistical Analysis; Page 8-10, Results, Fluoroscopy Time and Dose-Area Product sections; Page 10, Results, Procedural Duration section. |  |
|  | 13f | Describe any sensitivity analyses conducted to assess robustness of the synthesized results. | Page 6-7, Methods, Quality Assessment, Publication Bias, and Statistical Analysis; Page 10, Results, Procedural Duration section. |  |
| Reporting bias assessment | 14 | Describe any methods used to assess risk of bias due to missing results in a synthesis (arising from reporting biases). | Page 6-7, Methods, Quality Assessment, Publication Bias, and Statistical Analysis; Page 15, Limitations and Future Research. |  |
| Certainty assessment | 15 | Describe any methods used to assess certainty (or confidence) in the body of evidence for an outcome. | Page 6-7, Methods, Quality Assessment, Publication Bias, and Statistical Analysis; Page 15, Limitations and Future Research. |  |
| **RESULTS** | | |  |  |
| Study selection | 16a | Describe the results of the search and selection process, from the number of records identified in the search to the number of studies included in the review, ideally using a flow diagram. | Page 7-8, Results, Study Selection and Characteristics, paragraph 1; Figure 1. |  |
|  | 16b | Cite studies that might appear to meet the inclusion criteria, but which were excluded, and explain why they were excluded. | Figure 1, PRISMA flow diagram of study selection. | Cite studies that might appear to meet the inclusion criteria, but which were excluded, and explain why they were excluded. |
| Study characteristics | 17 | Cite each included study and present its characteristics. | Page 7-8, Results, Study Selection and Characteristics. |  |
| Risk of bias in studies | 18 | Present assessments of risk of bias for each included study. | Page 7-8, Results, Study Selection and Characteristics; Figures 2A–B and Figures 3A–B. |  |
| Results of individual studies | 19 | For all outcomes, present, for each study: (a) summary statistics for each group (where appropriate) and (b) an effect estimate and its precision (e.g. confidence/credible interval), ideally using structured tables or plots. | Page 7–12, Results, outcome-specific synthesis sections; Table 2; Figures 4A–7D. |  |
| Results of syntheses | 20a | For each synthesis, briefly summarise the characteristics and risk of bias among contributing studies. | Page 7-8, Results, Study Selection and Characteristics; Table 1; Figures 2A–B and 3A–B; Page 7–12, Results, outcome-specific synthesis sections. |  |
|  | 20b | Present results of all statistical syntheses conducted. If meta-analysis was done, present for each the summary estimate and its precision (e.g. confidence/credible interval) and measures of statistical heterogeneity. If comparing groups, describe the direction of the effect. | Page 7–12, Results, outcome-specific synthesis sections; Figures 4A–7D |  |
|  | 20c | Present results of all investigations of possible causes of heterogeneity among study results. | Page 8–10, Results, Fluoroscopy Time, Dose-Area Product, and Procedural Duration sections. |  |
|  | 20d | Present results of all sensitivity analyses conducted to assess the robustness of the synthesized results. | Page 6-7, Methods, Quality Assessment, Publication Bias, and Statistical Analysis; Page 10, Results, Procedural Duration section. |  |
| Reporting biases | 21 | Present assessments of risk of bias due to missing results (arising from reporting biases) for each synthesis assessed. | Not formally assessed; discussed in Page 8, Limitations and Future Research. |  |
| Certainty of evidence | 22 | Present assessments of certainty (or confidence) in the body of evidence for each outcome assessed. | Not formally assessed. |  |
| **DISCUSSION** | | |  |  |
| Discussion | 23a | Provide a general interpretation of the results in the context of other evidence. | Page 12–13, Discussion, paragraphs 1–5. |  |
|  | 23b | Discuss any limitations of the evidence included in the review. | Page 15, Limitations and Future Research. |  |
|  | 23c | Discuss any limitations of the review processes used. | Page 15, Limitations and Future Research, where limitations related to publication-bias assessment and heterogeneity exploration are discussed. |  |
|  | 23d | Discuss implications of the results for practice, policy, and future research. | Page 14-15, Discussion, final paragraph; Page 15, Limitations and Future Research; Page 15-16,Conclusions. |  |
| **OTHER INFORMATION** | | |  |  |
| Registration and protocol | 24a | Provide registration information for the review, including register name and registration number, or state that the review was not registered. | Page 4-5, Methods, Literature Search Strategy, where PROSPERO registration ID CRD420261345511 is reported. |  |
|  | 24b | Indicate where the review protocol can be accessed, or state that a protocol was not prepared. | Page 4-5, Methods, Literature Search Strategy. |  |
|  | 24c | Describe and explain any amendments to information provided at registration or in the protocol. | Page 6-7, Methods, Quality Assessment, Publication Bias, and Statistical Analysis. |  |
| Support | 25 | Describe sources of financial or non-financial support for the review, and the role of the funders or sponsors in the review. | Page 16, Declarations, Funding. |  |
| Competing interests | 26 | Declare any competing interests of review authors. | Page 16, Declarations, Conflict of interest. |  |
| Availability of data, code and other materials | 27 | Report which of the following are publicly available and where they can be found: template data collection forms; data extracted from included studies; data used for all analyses; analytic code; any other materials used in the review. | Page 6-7, Methods, Quality Assessment, Publication Bias, and Statistical Analysis; Page 16, Declarations, Data availability statements; Supplementary materials. |  |

*From:*  Page MJ, McKenzie JE, Bossuyt PM, Boutron I, Hoffmann TC, Mulrow CD, et al. The PRISMA 2020 statement: an updated guideline for reporting systematic reviews. BMJ 2021;372:n71. doi: 10.1136/bmj.n71. This work is licensed under CC BY 4.0. To view a copy of this license, visit <https://creativecommons.org/licenses/by/4.0/>
